# Current perspective on biological properties of plasmacytoid dendritic cells and dysfunction in gut

**DOI:** 10.1002/iid3.1005

**Published:** 2023-09-20

**Authors:** Xueran Guo, Chengwei He, Shuzi Xin, Han Gao, Boya Wang, Xiaohui Liu, Sitian Zhang, Fengrong Gong, Xinyi Yu, Luming Pan, Fangling Sun, Jingdong Xu

**Affiliations:** ^1^ Department of Clinical Medicine, Beijing An Zhen Hospital Capital Medical University Beijing China; ^2^ Department of Physiology and Pathophysiology, School of Basic Medical Sciences Capital Medical University Beijing China; ^3^ Department of Clinical Laboratory, Aerospace Center Hospital Peking University Beijing China; ^4^ Key laboratory of Carcinogenesis and Translational Research (Ministry of Education/Beijing) Peking University Cancer Hospital & Institute Beijing China; ^5^ Department of Clinical Medicine, School of Basic Medical Sciences Capital Medical University Beijing China; ^6^ Department of Laboratory Animal Research, Xuan Wu Hospital Capital Medical University Beijing China

**Keywords:** immune response, infection, intestinal mucosa, neoplasm, plasmacytoid dendritic cells, type I interferons

## Abstract

Plasmacytoid dendritic cells (pDCs), a subtype of DC, possess unique developmental, morphological, and functional traits that have sparked much debate over the years whether they should be categorized as DCs. The digestive system has the greatest mucosal tissue overall, and the pDC therein is responsible for shaping the adaptive and innate immunity of the gastrointestinal tract, resisting pathogen invasion through generating type I interferons, presenting antigens, and participating in immunological responses. Therefore, its alleged importance in the gut has received a lot of attention in recent years, and a fresh functional overview is still required. Here, we summarize the current understanding of mouse and human pDCs, ranging from their formation and different qualities compared with related cell types to their functional characteristics in intestinal disorders, including colon cancer, infections, autoimmune diseases, and intestinal graft‐versus‐host disease. The purpose of this review is to convey our insights, demonstrate the limits of existing research, and lay a theoretical foundation for the rational development and use of pDCs in future clinical practice.

## HIGHLIGHTS

- pDCs are a subset of dendritic cells, and transcription factors play a crucial role in the process.
- pDCs contribute to intestinal immune responses by generating interferon (IFN) and presenting antigens.
- pDCs guard the intestinal mucosa and protect against the invasion of microbiota and pathogens in the gut, playing a pivotal role in gut infection and colon cancer.

## Graphical abstract

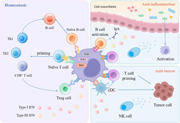

## 1 INTRODUCTION

The gastrointestinal immune system maintains its homeostasis mainly in a protective manner through the interaction of innate, adaptive, and innate‐like immune cells in different segments of the intestine.^1^ Dendritic cells (DCs), regarded as unrivaled essential antigen‐presenting cells (APCs), govern the immune system and connect innate information to specific adaptive responses,^2^, ^3^ safeguarding us against the dysregulation of homeostasis. They are heterogeneous and can be categorized into two types: conventional DCs (cDCs) and lymphocyte‐like plasmacytoid DCs (pDCs), both of which respond to sickness by releasing type I interferon (IFN, illustrated in Box 1). Langerhans cells and monocyte‐derived DCs are also considered to be DC subtypes.

Box 1 **Classification and nomenclature of interferons**

Interferons are categorized into three types based on their nucleotide sequence, interaction with certain receptors, chromosomal position, structure, and physicochemical properties.^4^ Type I IFN include α, β, ω, κ, ε, ζ, τ, δ, and ν, among which, we set our attention on IFN‐α and IFN‐β. Type II interferon is IFN‐γ and type III interferons include IFN‐λ1, IFN‐λ2, and IFN‐λ3. The IFNs, especially type I and type III IFN, highlight our health and disease by recognizing viral infections via innate immune sensors^5^ and guarding the host against tumors by signaling pathway activation.^6^

People have recently put a higher value on pDCs. It is commonly assumed that pDCs may rapidly and massively produce type I IFN and specialize in endosomal Toll‐like receptor (TLR) 7/9‐mediated recognition of viral and self‐nucleic acids.^7^ Thereby, they participate in a wide range of disorders, especially cancer, viral infections, and autoimmune diseases. Although little is known about the involvement of pDCs in gastrointestinal problems, it does bring up some unresolved issues concerning the treatment of intestinal ailments. It will also herald a new era of knowledge gathering and the identification of the role of pDCs in the digestive system.

Therefore, in this essay, we will focus on pDCs and analyze their development, properties, and subtype‐specific functions they have in the gut of pDCs in depth in the following sections.^8^

## 2 GENERAL BIOLOGICAL PROPERTIES OF PDCS: A COMPARISON OF DIFFERENT SUBTYPES OF DCS

DCs are classified into CD11c^+^ cDCs and CD11c^−/lo^ pDCs based on lineage and the differential expression of key transcription factors (TF).^3^ cDCs are divided into two subsets, interferon regulatory factors (IRF)8^+^ BDCA3/CD141^+^CLEC9a^+^ cDC1s and IRF4^+^BDCA1/CD1c^+^ cDC2s. This is based on several variables, such as the establishment of its unique transcriptional program development, phenotypic markers, and their divergent ability to stimulate CD8 and CD4 T cells,^8^, ^9^ while cDC2s comprise cDC2A and cDC2B.

Conflicting opinions exist on whether it is appropriate to categorize pDCs as DCs.^10^ Historically, pDCs were thought to be DC‐like APCs capable of secreting type I IFN, expressing certain TLRs, and eliciting T cell responses in both humans and mice.^11^ Recent studies have called into question the classification of pDCs, Ziegler‐Heitbrock et al. proposed that pDCs should be reclassified as innate lymphocytes due to their shared developmental trajectory with B cells, as well as their lack of classical functional properties, and gene expression profile as that conventional DCs have.^12^, ^13^ Thus, some researchers will henceforth refer to them as plasmacytoid cells (PCs)^14^ rather than DCs. Reizis et al., on the other hand, alluded to the fact that the categorization was premature.^15^

The debate is still ongoing. Therefore, we summarized the various viewpoints in this debate. The core is the heterogeneity of DCs, including subsets with different traits, tissue locations, developmental pathways, phenotypes, transcriptomes, and functions (depicted in Table 1).

**Table 1:** Comparison of biological characteristics between cDCs and pDCs.

	cDC1	cDC2	pDC	References
Morphology	Typical DC‐like, long dendritic extensions	DC‐like, long dendritic extensions	Round, lymphocyte‐like	[15]
Location (human)	Lymphoid organs and peripheral tissue	Lymphoid organs and peripheral tissue	Lymphoid organs and non‐lymphoid tissue	[15, 16]
Location (mouse)	lymphoid tissue, migratory/non‐lymphoid tissue	lymphoid tissue, migratory/non‐lymphoid tissue	lymphoid tissue, MLN, Peyer's Patch	[17]
Origin	Myeloid origin	Myeloid origin	Myeloid origin, lymphoid origin	[18]
Relative life scans	Short	Short	Long	[19]
Surface expression (mouse)	XCR1, CD11c, CLEC9A, CD24, CD205, CD8α (lymphoid‐resident) and/or CD103 (migratory)	CD103, CD11c, SIRPα, CD11b	CD11c^low^, PDCA1, Siglec‐H, B220, CD209, SIRPα, Ly6C, BST‐2, LAG‐3, CD8α, Ly49Q29	[8, 20]
Surface expression (human)	CD141, XRC1, CLEC9A, CADM1	CD1c/BDCA‐1, CD11c, CD11b, CD2, SIRPα, CD103a, FCER1	CD123/IL‐3Rα, CD303/CLEC4C/BDCA‐2, CD304, BDCA‐4, CD45RA, CD85k, CD85g, CD4, CD2, CD7, ILT7	[20, 21, 22]
MHC class II expression	Highly expressed by mature cDC1 but not immature cDC1	Highly expressed by mature cDC2 but not immature cDC2	Low level in steady state, upregulate upon activation	[15]
Main transcription factor expression	IRF‐8, Batf3, ID‐2	IRF‐4, Notch2, Klf4	IRF‐8, TCF4 (E2‐2), BCL‐11A, IRF‐7	[8, 18]
PRR expression (mouse)	TLR‐2, TLR‐3, TLR‐4, TLR‐9, TLR‐11, TLR‐12, TLR‐13, STING	All TLR except TLR‐3, TLR‐5, TLR‐11, TLR‐12, RLR, NLR, STING	TLR7, TLR9, TLR12, RLR, STING	[23]
PRR expression (human)	TLR‐1, TLR‐3, TLR‐6, TLR‐8, TLR‐10, STING	TLR2, TLR4, TLR5, TLR6, TLR8, TLR9	TLR7, TLR9, TLR1, TLR6	[15, 23, 24, 25, 26]
Chemokines/chemokines receptor	IL‐12, IL‐6, type III IFN	IL‐6, TNF‐α, IL‐23	Type I and III interferons, IL‐12, IL‐6	[16, 27]
Function in human	Antigen recognition and cross‐presentation to MHC class I, activate naïve T cells and IL‐6, production	Antigen presentation, activate naïve CD4^+^ T cells through IL‐12 production, express toll‐like receptor and product cytokine, IL‐6, TNF‐α, IL‐23 production	Producing IFN, sensing intracellular viral or self‐DNA and RNA mainly via TLRs, activating CD4^+^ T cells and CD8^+^ T cells	[8, 16, 18, 22]
Function in mice	Lacking cDC1s, Th2 responses	Treg induction, Th17 response	Producing IFN via TLRs	[20, 28, 29, 30]

### 2.1 Appearance, origination, development and location

The unique phenotype of pDCs is determined by their distinctive phenotype, including their appearance, origination, development, and location.

The moniker pDC refers to a combination of its DC‐like function and plasma cell appearance. However, unlike cDC, pDC has spherical plasma cells rather than dendrite‐like protrusions. Though pDCs develop a dendritic morphology after activation, cDCs are more capable of upregulating the expression of MHC‐II, co‐stimulatory molecules, and T cells than pDCs.^31^

Though pDCs and cDCs are thought to have originated from different progenitors in the past, recent research suggests that they seem to share ontogeny. pDCs can originate from lymphoid progenitors and myeloid progenitors, which may emerge from a common pool of CX3CR1^+^ hematopoietic progenitors.^15^ Their equivalent life scan reveals the same developmental kinetics.^21^ The development of pDC is frequently connected to the formation of lymphoid tissue‐resident cDC but not to that of inflammatory, monocyte‐derived DC. pDCs can differentiate into cDCs after activation. However, the mechanism of development of each phenotype differs: cDCs rely on *Irf8, Batf3, Id2, Nfil3*, and *Bcl6*, whereas pDCs primarily rely on the E2‐2 pathway. From an ontogenetic standpoint, sharing a common ancestor justifies pDCs membership in the DCs family. It also states that pDCs are descended from a common ancestor with cDCs.^32^

Following that, the majority of human pDCs spend their time in lymphoid organs and tissues; they can circulate via the lymphocinesia and account for 0.1%–0.5% of nucleated cells.^16^ Human cDCs have been detected in blood, lymphoid tissues including lymph nodes, tonsils, spleen, and bone marrow (BM), as well as non‐lymphoid tissues like skin, lung, intestine, and liver.^3^ Murine cDCs have been detected in lymphoid tissue such as the thymus, spleen, lymph nodes, and Peyer's patches (PP), as well as non‐lymphoid tissue.

Overall, there's a considerable difference between pDCs and cDCs in terms of appearance, development, and location, but this also highlights a correlation between the pDC and the cDC lineages, so we cannot make a blanket judgment.

### 2.2 Surface markers

pDCs are recognized as a distinct cell lineage due to their phenotypic cell surface markers and functional features.

#### 2.2.1 Surface markers of DCs in mouse and human

The markers of DCs in mice and humans are not identical. cDC1s in mice are rarer than cDC2s. The expression of CD45, CD135, CD11c, and MHCII are distinguished from that of cDCs.^14^ CD8, CD1c, signal regulatory protein α (SIRPα), C‐Type Lectin Domain Containing 9A (CLEC9A/DNGR‐1), and X‐C Motif Chemokine Receptor 1 (XCR1) are all expressed by murine cDC1s. CD4, SIRPα, and CD11b are markers for cDC2s.^14^ Interestingly, cDC2 lacks CD103 except in the intestine.^33^ Tcf4 (E2‐2), Ikzf1 (Ikaros), CLEC4C (BDCA‐2), SiglecH, mPDCA‐1/BST2 (CD317) are all expressed by pDCs.^16^

In humans, the surface markers are CD141/BDCA‐1, CLEC9A, cell adhesion molecule 1 (CADM1), B and T lymphocyte attenuator (BTLA) and CD26 in myeloid cDC1; CD1c/BDCA‐1, CD11c, CD11b, CD2, Fc epsilonR1 (FceR1) and SIRPα in myeloid cDC2; CD123 (IL‐3R),^34^ CD303/CLEC4C/BDCA‐2, CD304/NRP1/BDCA‐4, FCER1, ILT3/CD85k, ILT7/CD85g, DR6, XCR1, DNGR‐1 and CD4 in pDCs^3^, ^16^, ^35^, ^36^, ^37^, ^38^, ^39^ (Table 2). Besides, recently characterized antigens Fc epsilonR1 alpha, B and T lymphocyte attenuator (CD272), death receptor 6 (TNFRSF21/CD358) and CD300A are closely involved in the regulation of pDCs’ major physiological functions.^3^ As cDCs, pDCs lack additional lineage‐specific markers, including CD3, CD19, CD14, CD16, CD33, CD56, CD11b, and CD11c.^3^, ^52^

**Table 2:** The surface markers of human pDCs.

Surface markers	Functions	References
CD123 (IL‐3R)	Markers of pDC, characterize pDC, maintain pDCs alive, express of MHC‐II, CD80, CD86, CD40, produce TNF‐α, IL‐6, CCL4, CCL5, CXCL9, and CXCL10	[40]
BDCA‐2 (CLEC4C, CD303)	Suppress TLR7/9‐induced type I IFN production	[41, 42]
BDCA‐4 (NRP1, CD304)	Markers of pDC, characterize pDC, suppress TLR7/9‐induced type I IFN production	[42]
CD45RA	Typical pDC markers	[43]
CD4	Co‐receptor, upregulated through CpC stimulation	[28]
ILT3	Modulates type I IFN production	[28]
ILT7 (CD85g)	Downregulate type I IFN production	[44]
LAMP4 (CD68)	Tissue homing, promotes phagocytosis	[16, 28]
B7‐1 (CD80)	Co‐stimulatory, interact with T cells	[28, 45]
B7‐2 (CD86)	Co‐stimulatory, interact with T cells	[28, 45]
TNFRSF5 (CD40)	Co‐stimulatory, TD class switch recombination, anti‐inflammation	[16, 46]
FLT3 (CD135)	Interact for pDCs development	[18]
TLR‐7	Induction of type I IFN, type III IFN and pro‐inflammatory cytokines, viral detection	[44]
TLR‐9	Induction of type I interferons and pro‐inflammatory cytokines, viral detection	[44]
RIG‐I (DDX58)	Induction of type I interferons and pro‐inflammatory cytokines, detection of viral RNA upon infection	[28, 47]
MDA‐5 (IFIH1)	Induction of type I interferons and pro‐inflammatory cytokines, detection of viral RNA upon infection	[28, 48]
CCR2	Chemokine receptor, peripheral blood homing during homeostasis or inflammation	[43]
CCR5 (CD195)	Gut homing, promoting the migration from BM to the peripheral	[17]
CCR7 (CD197)	Peripheral LN homing	[49]
CCR9 (CD199)	Gut homing	[49]
PKR (EIF2AK2)	Innate immune response to viral infection	[16]
LY75 (CD205)	Promoting the binding of antigen and receptor, facilitating the manifestation of immune tolerance	[50]
αEβ7 integrin (CD103)	Gut homing, cellular intraepithelial morphogenesis and motility, viral detection	[51]
β2 integrin (CD18)	Gut homing, producing IFNα in response to TLR7 activation	[41]
α4β7 integrin (LPAM‐1)	Migration to gut and lymphoid tissue	[20]
P‐selectin (CD62P)	Gut homing	[16]
CXCR4 (CD184)	Chemokine receptor	[44]
CXCR3	Chemokine receptor	[44]
TNFRSF13B (CD267)	Inducing IgA through TI pathway	[16]
CD62L	Ag internalization for T cell presentation	[44]

Abbreviations: CXCL9, C‐X‐C motif chemokine ligand 9; CXCL10, C‐X‐C motif chemokine ligand 10.

#### 2.2.2 Shared surface markers between pDC and other DC subtypes

Some may contend that the surface markers justify pDC's lineage affiliation. However, it is challenging to identify if the shared marker expression is the consequence of other pathways or just lineage affiliation. The shared expression of IL‐7R/CD127 and Ly6D between pDC precursors and lymphocytes, for example, may be due to comparable E2 transcription factor activation.^16^

CD123 was formerly utilized to distinguish pDC; however, recent studies have revealed that CD123^+^ populations can also be detected in myeloid cDC‐like cells. This demonstrated the ability of pDCs to differentiate from myeloid cDCs in vitro.^53^ Furthermore, there can be ambiguity in prior research that requires additional confirmation.^3^, ^32^, ^54^

Nowadays, a population is defined by co‐expression of unique markers (e.g., AXL, SIGLEC1, SIGLEC6, and CD22/SIGLEC2) discovered by unbiased analysis. The non‐pDCs began to express myeloid cDC antigens, such as CD11c, CD33 (SIGLEC 3), and CX3CR1.^55^

pDC‐like cells, also described as CD56^+^ myeloid DCs,^56^ AXL^+^ Siglec6^+^ DCs (AS‐DCs), or pre‐DCs,^32^ were found in the BM and spleen at a frequency of 5%–10% of the mature pDCs, with high antigen‐presentation capacity and minimal of type I IFN production. They similarly did not respond to CpG‐B stimulation, but MHC‐II and the co‐stimulatory molecule CD86 were greater after CpG‐A stimulation.^32^

AS‐DCs were identified at the frequency of 2%–3% inside the original DC gate,^55^, ^57^ with shared surface marker IL3RA/CD123 and ITGAX/CD11C with pDC and cDC gene signatures. High expression of AXL and SIGLEC1 was related to elevated CD123, CD303, and CD141 and decreased HLA‐DR. Despite the fact that AS‐DCs shared markers with pDCs (IL3RA/CD123 and CLEC4C/CD303), their phenotype and function differed.^57^ AS‐DCs increased IL‐8 levels but were unable to induce type I IFN in response to TLR9 stimulation, whereas pDCs did the opposite.^57^

In mice, there were additional specialized pDC subtypes designated as transitional DCs (tDCs), which shared a phenotypic continuum between pDCs and cDC2s.^54^ CD11c^low^ tDCs exhibited a phenotype similar to pDCs, with higher levels of SiglecH, mPDCA‐1/BST2 (CD317), and B220, but a lack of CCR9 and Ly6D. CD11c^high^ tDCs matured closely resembling cDC2s, but activated differently from pDC‐like cells.^58^ Furthermore, gene detection validated the homology of mouse and human tDCs.^54^

During viral infection, tDCs shared developmental traits, essential transcription factor expression, and activities with pDCs.^54^ They were less capable than pDCs in producing type I IFN, but were better in promoting allogeneic T cell proliferation. The decreasing potential of pDCs in type I IFN generated upon stimulation was shown to be strongly connected to tDCs.^59^ Through a new mouse model based on intersectional genetics, it was postulated that pDCs might have inhibited this function in their neighbors to produce type I IFN, limiting excessive inflammation and the consequent immunopathological reaction.^58^

Newly formed DC subsets may be phenotypically and transcriptionally comparable to pDCs, with common surface markers reflecting transcriptional procedure and roles across phenotypes. Future research will undoubtedly be challenging, and advances in extracting a pure pDC, such as single‐cell RNA sequencing and a high‐dimensional single‐cell approach called mass cytometry (CyTOF), are essential.^54^

### 2.3 Functions

Owing to their unique biological properties, they play a variety of roles in human beings. cDCs are masters at antigen capture, processing, and presentation,^60^ while pDCs always play an integral role in secreting type I and III IFN.

#### 2.3.1 Main characters of cDCs

cDC1s are constantly engaged in antigen presenting and recognition. Once infected by viruses, they secrete type III interferon (IFN‐λ or IL‐29), while expressing high levels of TLR3 to detect viral RNA in dead cell debris internalized by cDC1s.^14^ Besides, due to the strong ability for antigen cross‐presentation via MHC class I to activate CD8^+^ T cells, murine cDC1s also express TLR11 and TLR12, stimulating the expression of IL‐12 and balancing the innate and adaptive immunity response.^61^, ^62^

cDC2s are essential for activating CD4^+^ T cells, expressing a wide range of TLR (TLR1, TLR2, TLR4, TLR5, TLR6, and TLR8) and secreting IL‐12, IL‐23, and IL‐6. cDC2s stimulate Th1, Th2, Th17, and CD8^+^ T cells in vitro and induce a variety of immunological responses in vivo.^3^, ^63^

#### 2.3.2 Main features of pDCs

**Figure 1:**
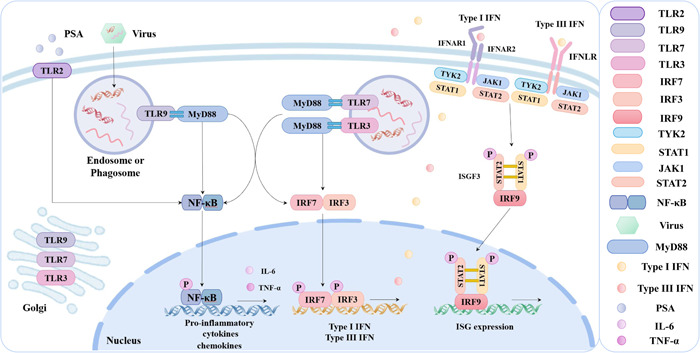
Summary of mechanisms by which pDCs producing type I IFN, type III IFN, and pro‐inflammatory cytokines. pDCs sense virus DNA and activate TLR9, TLR7, and TLR3 signaling in endosome or phagosome, which proceeds through signaling adapter molecules MyD88. MyD88 can activate NF‐κB to induce pro‐inflammatory gene expression and IRF3 and IRF7 to mediate type I IFN and type III IFN expression. Type I IFN and type III IFN secrete outside the pDC, and can combine with IFNAR and IFNLR receptors to further induce ISG expression. IFN, interferon; IFNAR, interferon‐α/β receptor; IFNLR, interferon lambda receptor; IL, interleukin; IRF, interferon regulatory factor; ISG, interferon‐stimulated gene; JAK, Janus kinase; MyD88, myeloid differentiation factor 88; NF‐κB, nuclear factor kappa‐B; pDC, plasmacytoid dendritic cell; PSA, polysaccharide A; STAT, signal transducer and activator of transcription; TLR, Toll‐like receptor; TNF, tumor necrosis factor; TYK, tyrosine kinase.

pDCs are specialized in response to virus infection with high production of type I and type III IFN, sensing pathogens through the nucleic acid‐sensing TLR. Mouse pDCs express the majority of TLRs except for TLR3, whereas human pDCs express significant TLR7 and TLR9 (Table 3), which mainly mediate the recognition of microbial pathogens,^68^ as well as TLR1, TLR2, TLR6, TLR11, and TLR12.^60^ TLR7 and TLR9 (depicted in Table 3) act a critical role by recognizing single‐stranded RNA and unmethylated CpG motif‐containing DNA and activating IRF7.^26^ pDCs predominantly produce type I IFN via PRR expression and activation, particularly signaling via the endosomal TLR9‐MyD88 pathway and activating NF‐κB^16^ (as shown in Figure 1). Besides, Ly49Q, abundantly expressed in pDC, is required for TLR9‐mediated type I IFN production and may protect pDC from cell death by regulating lysosome status.^69^ The human pDCs have also been shown to produce type I IFN in response to stimulator of interferon genes (STING) ligands.^25^

**Table 3:** Difference between TLR7 and TLR9 in recognizing microbial nucleic acids.

	TLR7	TLR9	References
Ligand specificity	Recognizes single‐stranded RNA (ssRNA) from viruses	Unmethylated CpG motifs in bacterial and viral DNA	[64, 65]
Cellular localization	Predominantly in pDCs; localized in the endosomal compartment	Expressed in various cell types; also localized in the endosomal compartment	[66]
Signaling pathways	MyD88‐dependent pathway, leading to the activation of NF‐κB and IRF7	TLR9 activates both the MyD88‐dependent and MyD88‐independent pathways, leading to the activation of NF‐κB and IRF7/IRF3	[67]
Role in autoimmunity	Implicated in the development of autoimmunity, recognize self‐nucleic acids under abnormal conditions		[64, 65]

The TLR/Th17 axis integrates innate and adaptive immune responses. TLR7 activation of human pDCs promoted Th17 cell differentiation from naive T cells,^70^, ^71^, ^72^ indicating that a TGF‐β‐rich environment on pDCs can trigger Th17 commitment.^73^ During chronic infection, viral single‐stranded RNA (ssRNA) stimulates TLR7, inducing and amplifying Th17 and enhancing the viral immune response.^74^, ^75^ In autoimmune disease, the activation of TLR7/9 in pDCs is shown to act as a promoting and perpetuating factor in disorders via recurrent stimulation by ssRNA/autoantibodies.^72^, ^76^, ^77^ Besides, as a primary sensor of the inflammatory response, pDCs release pro‐inflammatory and Th17‐skewing cytokines, leading to an IL‐23/Th17 divergence in psoriasis or eczema patients.^78^ Since Th17 are experts in promoting the recruitment of neutrophils,^79^ the activation of TLR7 on infiltrating pDCs might contribute to Th17 effector functions.^70^ The proteinase 3 (PR3)‐induced microenvironment facilitated the recruitment of pDCs and neutrophils, increasing pDC‐driven Th9/Th2 cell generation and altering immunological silence associated with the clearance of apoptotic neutrophils in patients with granulomatosis and polyangiitis.^80^

Furthermore, pDCs can uptake antigens, regulate peptide‐MHC complexes and antigen presentation, stimulate T cells, and express costimulatory molecules^10^ both in vivo and in vitro,^81^ whose effect is lower than cDC. During microbial infections, pDCs can inhibit the CD4^+^ T cell responses while participating in the CD8^+^ T cell responses in vivo.^82^ pDCs can attenuate autoimmune diseases, such as experimental autoimmune encephalomyelitis, by presenting MHC II‐mediated antigens (Ag) to IL‐17‐ and IFN‐γ‐secreting effector CD4^+^ T cells in secondary lymphoid tissue and by promoting a selective expansion of Ag‐specific forkhead box P3 (Foxp3^+^) Treg cells.^81^ When used in organ transplantation, pDCs are able to interact with Treg cells, acquire alloantigen in the allograft, tolerate pDCs transfer to peripheral lymph nodes through blood, promote peripheral Treg cell development, and induce tolerance.^83^ Additionally, pDCs are impacted indirectly by blood‐borne apoptotic cells and are implicated in Treg stimulation/generation.^84^ This may correlate to suppressing immune responses to self‐antigens of apoptotic cells and maintaining immune tolerance.

In the mucosal immune system, pDC contributes to the induction of oral tolerance and suppression of delayed‐type hypersensitivity (DTH) by affecting both CD4^+^ and CD8^+^ T cell responses as well as increasing pDCs’ amount.^85^, ^86^, ^87^ Oral tolerance was initially mediated by a high proportion of environmental Ag‐specific T cells rather than regulatory CD4^+^ T cell.^86^, ^88^, ^89^ They may suppress specific T cell priming mediated by CD4^+^ Treg cells with the same Ag in lymphoid organs during the subsequent stage of oral tolerance.^90^ They share similar tolerogenic qualities, but their tolerogenic potential may be influenced by the local microenvironment in vivo.^88^, ^89^ This gave way to the therapeutic approaches targeting pDC in intestinal inflammatory diseases, transplantation tolerance, and DTH reactions.

To further distinguish the functions and general biological characteristics of pDCs from those of cDCs, we analyzed the two types of cells, with the detailed information summarized in Table 1. There is still considerable disagreement about whether pDCs should be classified into DCs, and single‐cell‐level analysis between cDCs and pDCs is required in the future.

## 3 THE DEVELOPMENT PROCEDURE AND MULTIFACTOR REGULATION OF PDCS

**Figure 2:**
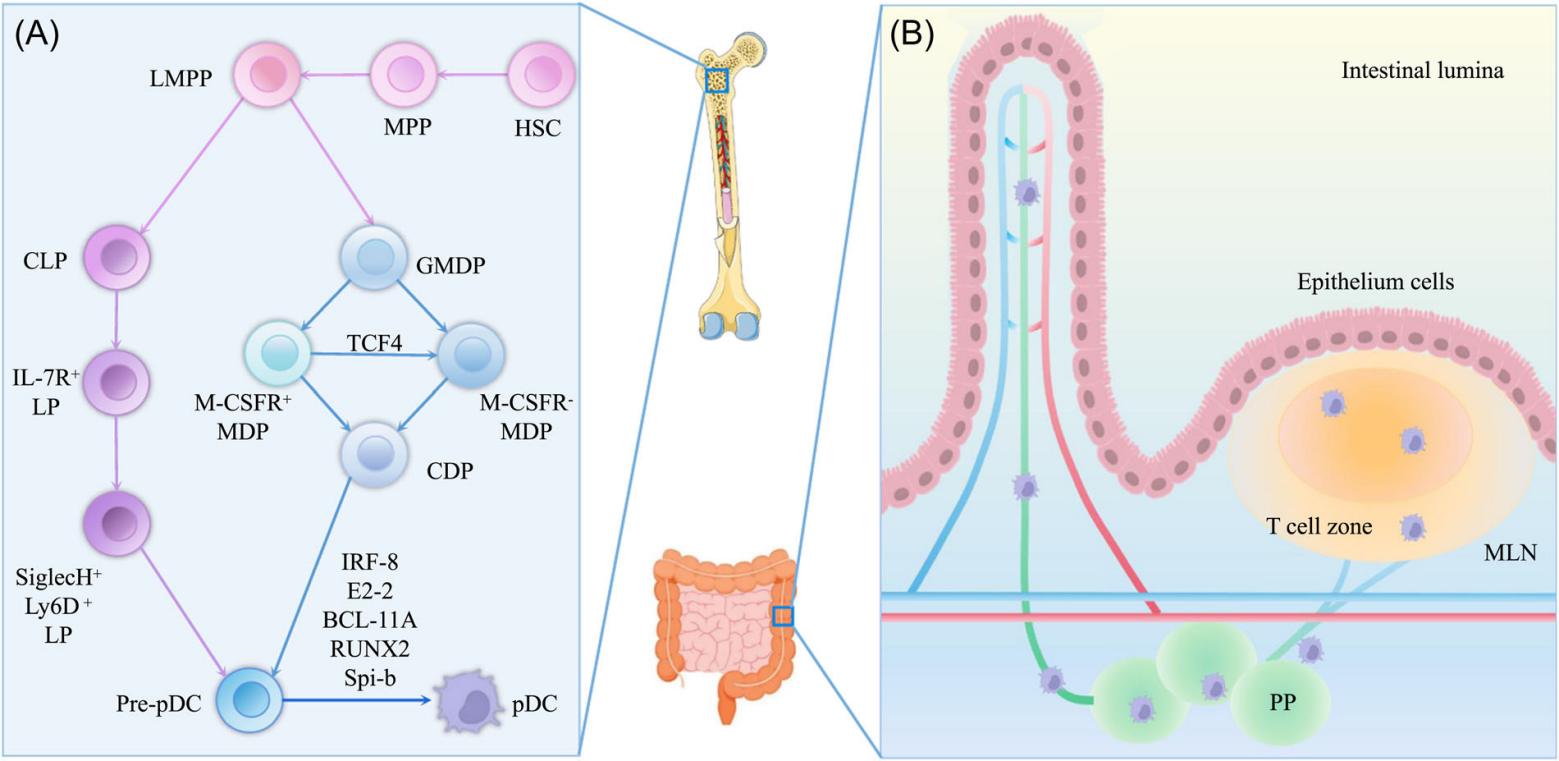
Pattern of pDCs development from bone marrow (A) to blood and then to the gut (B). (A) In the bone marrow, there are two paths for pDCs development after generating from HSC to MPP and then to LMPP. One of the pathways is that MDP generates M‐CSFR^+^ CDPs and M‐CSFR^−^CDPs, which subsequently form CDPs. Another way is for LMPP to evolve into CLP, which generates IL‐7R^+^ LP and eventually SiglecH^+^ Ly6D^+^ LP. Both paths eventually converge to form pre‐pDCs, which later give rise to pDCs. (B) pDCs migrate primarily through lymph nodes to MLNs and PPs, eventually settled in the gastrointestinal tract. CDP, common DC progenitor; CLP, common lymphoid progenitor; GMDP, granulocyte–macrophage DC progenitor; HSC, hematopoietic stem cell; IFN, interferon; LMPP, lymphoid primed multi‐potent progenitor; M‐CSFR, macrophage colony‐stimulating factor receptor; MLN, mesenteric lymph node; MDP, macrophage DC progenitor; MPP, multipotent blood progenitor; pDC, plasmacytoid dendritic cell; PP, Peyer's patch.

As mentioned above, the origin of pDCs led to several controversies. Nevertheless, data presently suggest that pDCs are produced from both myelogenous‐macrophage DC progenitors (MDPs) and lymphoid‐primed multipotent progenitors (LMPs) derived from BM and lymphoid cells.^20^ Both pDCs and cDCs originate from a common hematopoietic progenitor and differentiate through the FMS‐related tyrosine kinase 3 (FLT3L)‐induced signaling pathway.^91^ However, they diverge down a distinct pathway, requiring the expression of pDC‐specific transcription factors. Two myeloid genesis pathways are involved in pDCs' development (depicted in Figure 2A).

### 3.1 Two main ways of the development of pDCs from BM

The development of pDCs involves different stages and factors.^20^ After initial lineage commitment, pDCs undergo a rigorous differentiation program to implement functional integrity.

On the one hand, myeloid MDPs generate common DC progenitors (CDP), which give rise to pDCs.^20^, ^92^ CDPs are precursor cells with cDC and pDC potential, with M‐CSFR^−^CDPs having a higher pDC potential than M‐CSFR^+^ CDPs. A subgroup of CDPs can give birth to CCR9high^+/+^ pDCs, which can develop into pDCs in an E2‐2‐dependent manner.^20^, ^93^

pDCs, on the other hand, can be also derived from common lymphoid progenitor (CLP). Then it requires type I IFN signals for FLT3 upregulation.^20^ It has been found that an abundant proportion of pDCs expresses recombination activation genes (Rag1/Rag2) and undergoes the rearrangement of immunoglobulin DH‐JH, which only transiently expressed in the pDC lineage, and RAG1 expression and Ig rearrangement are mainly found in CLP‐derived pDCs^20^, ^94^ as illustrated in Figure 2A.

pDCs fully mature in the BM and migrate to the thymus and all secondary lymphoid organs via the bloodstream under steady‐state conditions, with the aid of RUNX2 and CCR5.^95^, ^96^ They primarily reside in and recirculate through lymphoid organs. Importantly, studies indicate that the distinction between cDCs and pDCs may occur at an early stage.^93^

### 3.2 The crosstalk between pDCs and cytokines

**Figure 3:**
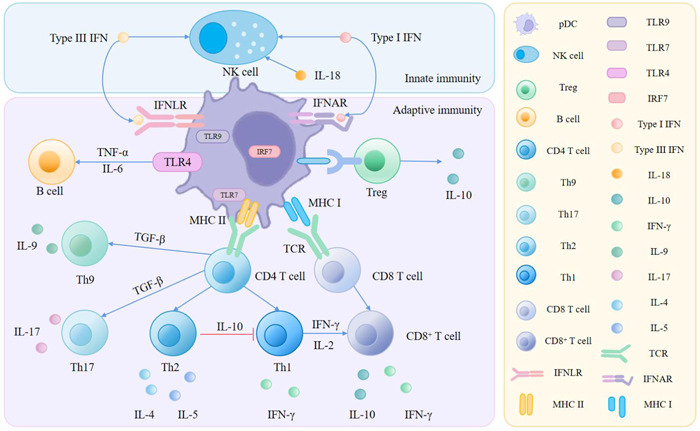
Multiple pathways play a role of pDCs in maintaining homeostasis through the immune system. In innate immunity, pDCs can activate NK cells by producing type I IFN, type III IFN, and IL‐18. In adaptive immunity, after TLR7/9 activation, pDCs can produce type I IFN and type III IFN. They can also interact with CD4^+^ T cell and CD8^+^ T cells, which then induce Th1, Th2, Th9, Th17, and CD8^+^ T cell‐producing cytokines. In addition, they promote B cell and Treg cell induction of IL‐10. IFN, interferon; IFNAR, interferon‐α/β receptor; IFNLR, interferon lambda receptor; IL, Interleukin; IRF, interferon regulatory factors; pDC, plasmacytoid dendritic cell; TGF‐β, transforming growth factor‐β.

The mechanism of pDC development is not completely deciphered. Murine models are always used to research the mechanism of human DC, since mouse and human pDCs share the same features in their transcriptome and developmental pathways. However, species differences restrict its capacity to accurately mimic the biology of human DCs,^97^ including distinct surface markers, transcription factors (as summarized in Table 4), and expression patterns of TLRs (Figure 3 and Table 3).

**Table 4:** Transcription factors and miRNAs involved in the regulation of human pDC development.

Classification	Regulating factors	Functions	References
Transcription factors	E2‐2 (TCF4)	Downregulate the expression of TLR10, Siglec‐6, ID2, contribute to the species‐specific difference	[60, 98]
	ZEB2	Repress Id2, determine the fate of pDCs	[99, 100]
	IRF8	Repress the pDC gene expression	[101]
	RUNX2	Induce IRF7 expression, upregulate type I IFN production	[102]
	IRF4	Determine the fate of pDCs	[3]
	IRF5	Activate CpG‐B‐triggered gene, upregulate type I interferon	[41, 103]
miRNAs	miR‐146a	Impair TLR‐mediated maturation, up‐regulate CD40, CD80, CD86, HLA‐DR, CCR7, IL‐6, and IFN‐γ	[2, 104]
	miR‐126	Improve pDC‐mediated activation, migration, release IFN‐γ, target the mTOR pathway	[2, 105, 106]
	miR‐29b	Regulate pDCs apoptosis and cell survival	[2, 107]
	miR‐29c	Regulate pDCs apoptosis and cell survival	[2, 107]
	miR‐22	Target Irf8 mRNA, induced in progenitor cultures by GM‐CSF	[28, 108]
	miR‐155	Express after TLR7 signaling and upregulate type I IFN production	[105, 109]
	miR‐155*	Express after TLR7 signaling and upregulate type I IFN production	[105, 109]

To begin with the distinct surface markers, they are marked by CD11c, Siglec‐H, BST2 (CD317), CD45R (B220), CD45RA, Gr‐1, Ly‐6C, and Ly49Q in mice.^20^ Human pDCs are marked by CD303 (BDCA2), CD304 (BDCA4), ILT7, CD123 (IL‐3R), CD45RA and CD4 (Table 2). Moreover, mutation of TCF4 or IRF8 also leads to pDCs defection.^98^

#### 3.2.1 Transcription factors inducing the generation of pDCs

Transcription factors of murine and human pDCs vary. Murine pDCs are influenced by E2‐2/TCF4, IRF8, B cell lymphoma 11a (Bcl11a), ZEB2, and Spi‐B. E2‐2/TCF4, ZEB2, IRF8, and IRF4 are all involved in the development of human pDCs.

To start with, the basic helix–loop–helix transcription factor (E protein) E2‐2/Tcf4 has previously been identified as a critical and particular regulator in both mice and humans,^98^ modulating pDC function in a species‐specific manner. The lowered expression of the TLR pathway explains why transcription factor E2‐2 increases the formation of pDC as well as the IFN response to unmethylated DNA. It can also directly activate pDC‐enriched genes to upregulate IFN‐α production in response to CpG in humans,^60^ including transcription factor Spi‐B, IRF8, IRF7, TLR9, TLR7, RUNX Family Transcription Factor 2 (RUNX2), and class II major histocompatibility complex transactivator,^30^, ^63^, ^98^, ^110^ while downregulating the expression of TLR10 and Siglec‐6.^60^

Constitutive high expression of Spi‐B contributes to transactivating the type I IFN promoters in collaboration with IFN regulatory factor 7 (IRF‐7), TLR7/9 induced type I IFN production in both human and mouse pDC.^26^ Spi‐B positively regulates the pDC lineage by promoting early commitment and enhanced survival.^3^ In Spi‐B^−/−^ mice,^26^ the amount of BM pDC was decreased, and the expression of pDC‐specific genes profile attenuated, whereas peripheral pDCs were increased, demonstrating the role of Spi‐B in pDC development. The fundamental mechanism of transcription factors that stimulate the development and specification of pDC remains unknown.

#### 3.2.2 Transcription factors suppressing the generation of pDCs

ID2, a DNA‐binding inhibitor discovered to control the balance of both marine and human pDC growth, suppresses the generation of pDC from CDPs^20^ by negatively regulating E2‐2.^63^, ^110^ ID2 is repressed by zinc‐finger E‐Box Binding Homeobox 2 (Zeb2), a zinc‐finger homeodomain transcription factor, and Myeloid Translocation Gene 16, an ETO family protein. Previous research demonstrated that the expression of Id2 and the absence of Zeb2 determine the ratio of cDC1 and pDCs^99^ and incomplete abrogation of pDC development caused by CD11c‐Cre‐driven Zeb2 deletion,^99^ indicating that Zeb2 is required in pDC development and regulates the fate between pDC and cDC1 with the help of *Id2*.^98^, ^100^

Recent research has discovered that leukemia inhibitory factor (LIF) inhibits pDC activity and development.^111^ *Lifr* mRNA was reported to be substantially expressed in pDCs from spleen and skin‐draining lymph nodes and spleen cells, and sorted pDCs produced high levels of IFN‐α following CpG‐A by evaluating the Immunological Genome Project. These findings indicate that pDC can express LIFR, which in turn inhibits pDC activities. As valued by RT‐PCR, stimulation of pDCs with LIFR inhibited type I IFN, TNF, and interleukin‐6 (IL‐6) responses to CpG, but induced expression of STAT3, Bcl3, and Id2, which in turn inhibited type I IFN and nuclear factor kappa‐B (NF‐κB) signaling.

In brief, pDCs development is dependent on the coordinated activity of several transcription factors to facilitate their differentiation, and the inhibitory transcription factors are crucial for their regulation. Nonetheless, there is still debate on the origin and lineage affiliation of pDCs. Clonal tracking of HSCs and CX3CR1^+^ progenitors in steady‐state hematopoiesis revealed common development pathways between pDCs and cDC1s, but not cDC2s or B cells.^112^ However, due to the technical limitation of the currently utilized clonal barcode readout, there was an overrepresentation of barcodes unique to a single cell type. Furthermore, the analysis of DCs development in inflammatory states remains uncovered.

#### 3.2.3 miRNAs

miRNA has been found to regulate pDC development^2^ (as depicted in Table 4). Inhibiting miR‐221^105^, ^113^ in BM‐derived DC progenitors led to the differentiation of pDC; high expression of miR‐126 in both humans and mice^105^, ^106^ and miR‐146a in humans^114^ resulted in pDCs survival. MiR‐126 deficiency reduces pDC‐mediated activation, migration, and IFN‐γ releases after TLR activation with CpG‐A.^115^ Increasing miR‐146a expression, which is induced by TLR ligation (TLR7/9), inhibited key components of the NF‐κB pathway to impair TLR‐mediated maturation, as well as the upregulation of CD40, CD80, CD86, HLA‐DR, and CCR7 molecules and the production of IL‐6 and IFN‐γ, thereby inhibiting pDC‐induced allogeneic T‐cell responses.^104^ Overexpression of miR‐146a, on the other hand, may trigger apoptosis in pDC, since it targets IL‐1 receptor‐associated kinase 1 and blocks TLR‐induced nuclear factor‐jB activity, leading to downregulation of antiapoptotic genes.^104^ Furthermore, miR‐29b and miR‐29c also include apoptosis and cell survival of pDCs.

These findings implicate a complicated transcriptional network in the development of pDCs to ensure their functionality (as shown in Table 4). To promulgate their role in various diseases and immune responses, further research is required to fully understand the multifactor regulation and development of pDCs, including epigenetic processes and regulatory factors.

## 4 GUT‐ASSOCIATED PDCS AND INTESTINAL IMMUNE HOMEOSTASIS

According to the studies,^116^, ^117^ pDCs aggregate in the inflamed colonic LP by integrating the gut‐homing receptors and play a crucial part in the development of acute colitis by regulating intestinal inflammation. The microenvironment has a profound impact on the features of intestinal pDCs, including the abundance of commensal bacterial metabolites and epithelial cell‐derived soluble substances.

### 4.1 Functional location of pDCs in the gut

The intestinal mucosa is considered to be the body's first line of defense since it is the largest mucosal tissue in the human body.^118^ Intestinal immune compartments comprised two inductive parts: the intestine‐draining mesenteric lymph nodes (MLN) and the gut‐associated lymphoid tissues (GALTs).^119^ The latter include PPs and isolated lymphoid follicles, under the monolayer epithelium. DCs are located in GALT, cDCs are located in LP, pDCs are located in LP, PP, and MLN, while monocyte‐derived DCs are located in LP and PP^120^ (detailed information depicted in Figure 2B).

Before being stimulated by the pathogen, pre‐pDCs migrate from BM to the intestinal epithelial layer,^49^ and then into the lumen in the steady state. Expression of CCR7 (CD197) and CCR9 (CD199), as well as their corresponding ligands CCL19 (MIP‐3β), CCL21 (6Ckine), and CCL25 (TECK),^121^, ^122^, ^123^ can be able to present self‐antigen and promote immune tolerance.^17^

Multiple models have corroborated the development of pDC into particular tissues via homing receptors. Gut‐associated pDCs express β2‐integrin (CD18), α4β7 integrin (LPAM‐1), P selectin (CD62P), and CCR9 (CD199), rather than CCR7 (CD197) in contrast to intestinal T and B cells. Although the majority of GALT‐associated leukocytes, such as subsets of T cells and cDCs, have been shown to express α4β7, CCR9, and/or CD103, both MLN and siLP pDCs, as well as splenic pDCs, have been shown to exhibit these gut‐homing receptors at various levels.^17^ Wherein CCR9 is essential in both the steady and inflammatory states, which typically occur in the small intestine and rarely in the colon.^124^ Deficiency of CCR9^+^ pDCs induced ileitis exacerbation.^17^ Adoptive transplantation of pDCs from wild‐type mice into CCR9^−/−^ mice restores normal migration.^49^ These results testify that CCR9 is a critical facet in the migration of pDCs and the maintenance of immunological homeostasis.

Unique expression patterns of gut‐homing receptors of cell adhesion molecules and chemokine receptors may represent the development and localization of tissue‐resident pDCs, providing an entirely novel avenue of investigation.

### 4.2 Gastrointestinal immune system and DCs

The gastrointestinal immune system is the most significant and complex part of the immune system, as the gastrointestinal tract is constantly exposed to an enormous variety of foreign materials that might be either harmful or beneficial to the organism. The gut harbors hundreds of millions of intestinal bacteria, which are crucial in controlling the intestinal mucosal barrier and in the research process.^125^

There are two types of immunity: adaptive immunity and innate immunity, and DCs are the bridge between these two immunities. T cells and B cells are prominent adaptive immune subtypes, whereas DCs with distinct roles belong to the innate immune system.^126^ Innate immunity, which includes the mucosal barrier, is the body's initial line of protection against toxins and pathogenic microbes. DCs operate as sentinels, identifying the presence of invading pathogens via numerous pattern recognition receptors and secreting several cytokines to induce inflammatory responses, after which they activate primary T‐cell responses.^3^ In the gut, DCs take up antigens by using specialized surface receptors and then transfer the antigen information to the MLNs. Inducing Tregs activation, they protect the relative stability of the intestinal environment^120^ and initiate cellular and humoral immune responses, as well as protect against infectious illnesses or malignancies.

The significance of pDCs in the gastrointestinal immune system has been revealed recently, but seldom do we figure out the explicit mechanism of pDCs. In the following sections, we will summarize the major role of pDCs in the gut (as illustrated in Figure 3).

### 4.3 Intestinal mucosal immune and pDCs

pDCs play a role in gut immune homeostasis by balancing immune regulation and inflammation. The role of pDC in the mucosal immunological barrier has received increasing attention in recent years.

pDCs are necessary for the digestive tract, particularly in GALTs, which are disproportionately abundant in the epithelium (intraepithelial, IE) and lamina propria (LP) compared to other tissues in the steady state.^49^ GALT‐associated pDCs appear to have adopted a unique phenotype,^127^ with diminished IFN‐α production when stimulated with CpG DNA,^128^ but the ability to promote Th17 and Treg differentiation activated with TLR agonists.^129^ They maintain the intestinal environment's homeostasis, defend against virus and toxin invasion, and suppress the onset of inflammation via the interaction of immune cells with the microenvironment.^130^

#### 4.3.1 Type I IFN production

As IFN‐producing cells, pDCs are believed to have little pivotal function in the intestinal mucosa. However, their role is slightly important in intestinal immunity. Functional pDCs and low‐level expression of type I IFN by stromal cells, however, are crucial for maintaining gut homeostasis in response to commensal bacteria.^17^ In MLN, stromal cells only expressed type I IFN in the steady state due to the TLR‐dependent recognition of commensal bacteria.

CD4^+^‐pDC is the primary source of IFN‐α and TNF‐α production compared with uninfected CD4^−^‐pDC.^117^ During the acute phase of infection, more than 80% of pDCs in the intestinal mucosa were produced by CD4^+^, and the responses of IFN‐α and TNF‐α, as well as TLR7/8 activation, were considerably higher than in naïve mice.

#### 4.3.2 Antigen presentation

**Figure 4:**
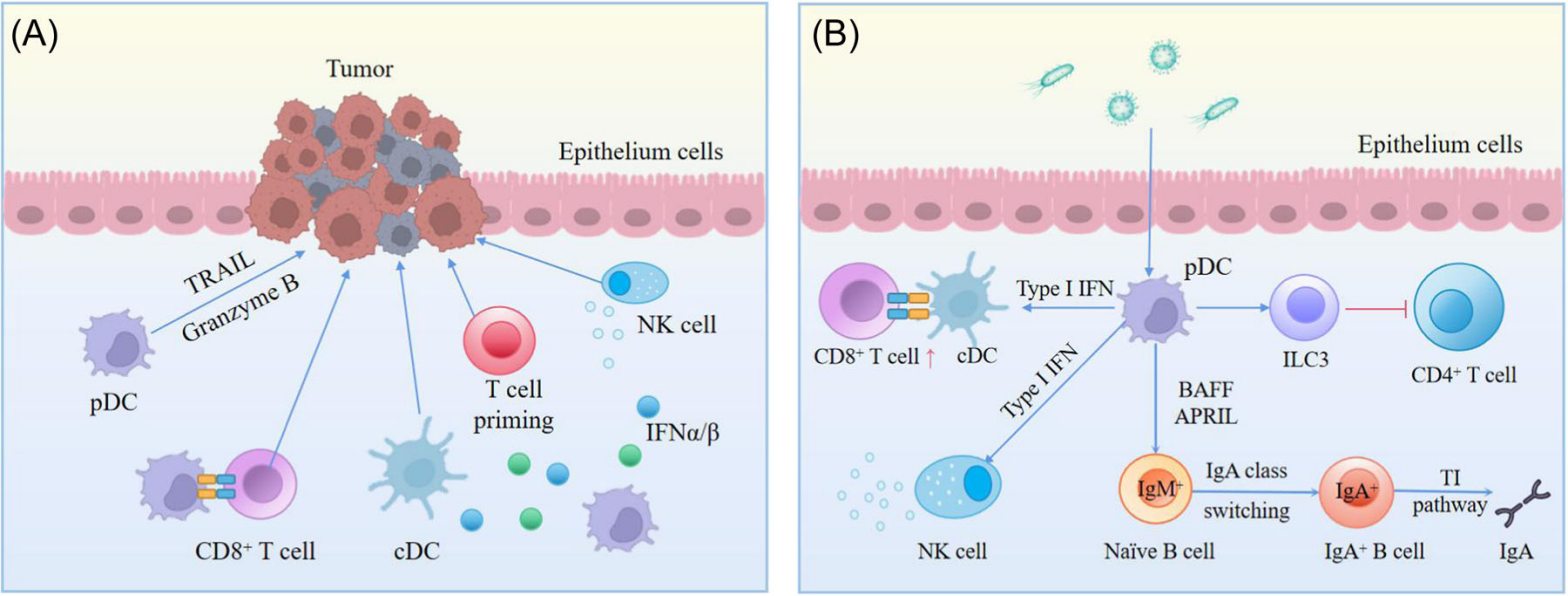
Diagram depicting the underlying mechanism of pDC activation towards tumor and inflammation in the gastrointestinal tract. (A) pDCs can directly secrete IFNα/β, and participate in antitumor activities through TRAIL‐dependent mechanisms and granzyme B secretion. They can also interact with CD8^+^ T cells, NK cells, cDCs, and T cells to protect against tumor. (B) To anti‐inflammation, pDCs in MLNs or PPs can release type I IFN to promote the interaction between CD8^+^ T cells and cDCs and activate NK cells. They also help to increase IgA by flipping naïve B cells promote. They can also interact with ILC3 and suppress the CD4^+^ T cell response. APRIL, TNF ligand superfamily member 13 (TNFSF13, APRIL or CD256); BAFF, B cell activating factor; cDC, conventional dendritic cell; IFN, interferon; pDC, plasmacytoid dendritic cell; TRAIL, TNF‐related apoptosis‐inducing ligand.

CD103^+^ DCs, which are involved in the maintenance of gut homeostasis, can induce immune tolerance to intestinal antigens and promote protective immune responses. pDCs can increase Th17 cell antibacterial activity while also inducing Treg cells under steady‐state circumstances.^17^, ^131^ pDCs are most likely involved in mucosal T‐cell‐independent (TI) routes for IgA induction,^132^ as well as T‐cell‐dependent pathways for B cell IgA acquisition. IgA has a modest affinity for TI pathways, and it may inhibit the attachment of commensal bacteria to intestinal epithelia without neutralizing them.^133^ pDCs of the MLNs and PPs representative GALT, as indicated in Figure 2B, contribute to naïve B cells inducing TI IgA class switch recombination, which has a considerably greater effect than cDCs. IgA production was dependent on TNF ligand superfamily member 13 (TNFSF13, APRIL, or CD256) and B cell activating factor (BAFF), which were related to stromal cell‐derived type I IFN signaling under steady state, as described in Figure 4B.^132^

Interestingly, pDCs appear to be dispensable for noninfectious IgA responses via TI pathways.^134^ Since the frequency and quantity of dividing cells of proliferating NP‐specific B cells were not affected by the pDC deletion after a 4‐day vaccination with NP‐Ficoll in a mouse model. Surface IgA has been identified in PP rather than in the spleen or MLN of BDCA2‐DTR^+/−^Ly5.1^+/−^ mice or wild‐type littermates. The evidence hints that pDCs are unimportant in terms of B‐cell division and IgA class switch recombination. Further evaluations of the antigen‐presenting capacity of pDC are warranted.

#### 4.3.3 Age‐related functional alterations

Interestingly, the functional alterations of pDCs in intestinal mucosa are age‐related.^123^ The proportion and absolute amount of CD3 intestinal IE cells and pDC were much lower in elderly mice than in young animals, as validated by RT‐PCR. *CCL2* and *IL‐6* expression was found to be elevated in aged mice, while *CCL25* mRNA and protein levels were considerably lowered, which was consistent with a drop in the number of pDCs accompanied by a decrease in the number of cells migrating in the LPS‐treated model. In further analysis, the expression of Ly6C^+^, B220^+^ pDC in the cell development center of Flt3L‐stimulated mobilization of pDC from splenocyte preparations was found to be more heterogeneous in the old cell compared to the young CFSE‐labelled pDC population, and the expression of CCR9 was found to be lower in the old cell compared to the young CD45.1^+^ pDC population. The aforesaid findings demonstrate that pDCs vary with age, which is connected to alterations in the elderly's intestinal microenvironment with decreased expression of CCL25 and pDC migratory capacity, implying a possible linkage in the etiology of gastrointestinal disorders in the elderly.

As a result, pDCs may serve a purported role in controlling gut mucosal immune homeostasis via their unique mode of action. In the meantime, they are active sentinels of colonic inflammation and/or microbial dysbiosis.^131^ Gut immunological homeostasis is maintained by the interaction between host immunity and intestinal factors, and pDCs contribute to this maintenance.

## 5 GUT‐ASSOCIATED PDCS AND DISEASE

Gut‐associated pDCs manifest a complex and context‐dependent involvement in a number of human disorders, including colorectal cancer, gut infection, IBD, and immune‐mediated diseases.^3^, ^16^ We emphasize the sensor signaling regulation of gut‐associated pDCs in gastrointestinal lymphoid tissue and briefly discuss its practice in digestive system disorders as depicted in the following sections.

### 5.1 Colorectal cancer

Cancer of the digestive system, specifically colon cancer, is associated with various clinicopathological characteristics. There is mounting evidence that the immune system may prevent the onset, progression, and metastatic diffusion of colorectal cancer (CRC).^135^ The host immune system can initiate an immune response against colon cancer cells, but tumor cells may utilize different strategies to avoid detection.^136^

pDCs have been implicated in the colonic tumor microenvironment,^23^ but innate lymphoid cells (ILCs) can sustain mucosal integrity by generating powerful cytokines.^137^ Upon research evidence, the complexity of pDCs in tumors corresponds with various clinicopathological characteristics in colon cancer, including clinical stages, progression‐free and overall survival, and prognosis. But in fact, little is known about pDCs’ possible function in colon cancer and their impact on patients. Thus, this section will outline the role of pDCs in the microenvironment of colon tumors.

#### 5.1.1 pDCs associated with the pathological stage, survival, and prognosis

The amount of pDCs is connected to the pathological and clinical stages of the tumor.^135^ The proportion of pDCs grew from proximal to distal, and the fraction of pDCs increased with the growth of the pathological stages of the tumor. The frequency of ILC3 and pDCs in the tumor‐like microenvironment was negatively related using RNA‐Seq, suggesting the gene pDCs expressed may be involved in tumor growth or inhibition.^138^ For instance, the upregulation of *ARHGAP4, HSPD1, HNRNPA2B1, UBAP2L, STAG1, TUBB, GPX2, CD44, PEBP4*, and *CD274* were associated with the progression, whereas the downregulation of *SNAP23, PTPRE, RPS13*, and *OGT* were associated with the tumor suppression.^135^ A patient population cohort analysis further indicated that the higher density of infiltrating pDCs, the better the overall survival of patients with colon cancer.^139^

#### 5.1.2 pDCs associated with the localization in tumor microenvironment

Colon‐cancer‐infiltrating pDCs may be closely related to tumor microenvironment localization.^139^ A proportion of IRF7^+^ pDCs, indicating an activated phenotype, were located in the neighborhood of granzyme B‐expressing CD8^+^ T cells in tumor stroma regions. Furthermore, pDCs were a crucial component in colon cancer‐associated tertiary lymphoid structures, displaying a nuclear IRF7 expression and being near CD4^+^ T cells.

In addition, the expression of *CLEC4C* by pDCs involved in ligand internalization and antigen presentation,^140^ is correlated with the patient survival. Multiple fluorescent labeling revealed that the spatial distribution of various T cell subtypes adjacent to the pDCs were CD8^+^ T cells, and the frequency of prolonged progression‐free survival was considerably higher in patients with colon cancer who had pDCs located close to CD8^+^ T cells. Therefore, the co‐localization of pDCs and CD8^+^ T in the tumor microenvironment may influence the regulation of tumor growth.^139^, ^141^

#### 5.1.3 Immunoreaction of pDCs in colon cancer

Gut‐associated pDCs can participate in antitumor immunity responses via innate and adaptive immunity, which regulates colon cancer growth and predicts prognosis. It has been shown that pDCs play a critical role in the suppression of colorectal cancer growth by influencing infiltrating myeloid‐derived suppressor cells (MDSCs).^142^ The deletion of TLR3 and TLR7 in mouse increase MDSCs but reduce pDCs, which are responsible for the aggravation of colitis‐associated colon cancer.

pDCs modulate the immune response in the tumor microenvironment, resulting in patient prognosis. Though it is yet unclear how pDCs directly or indirectly boost antitumor immunity, we can get a glimpse of the potential processes. They are believed to directly produce optimum cross‐priming and CD8^+^ T cell immunity, while also enhancing the activity of NK cells and T cells, and cDCs collaborate indirectly via the modulation of type I IFN^143^ (Figure 4A).

pDCs induce CD8^+^ T cells to secret IL‐10 and IFN‐γ after being loaded with immune complexes,^144^ which may lead to a predominantly Th2 response and also have cross‐presentation to CD8^+^ T cells in vitro.^145^, ^146^ Inactive pDCs exhibit low levels of MHC class I molecules (MHC‐I), class II, and even undetectable CD80 and CD86, which can be significantly expressed to gain the capacity to activate T cells.^147^ In response to tumor immunity, pDCs may cross‐present antigens and successfully activate antigen‐specific CD8^+^ T‐cell responses in both mice and humans, either via the TLR route or directly targeting the antigen.^148^

Both humans and mice pDCs have been proven to directly lyse tumor cells through TNF‐related apoptosis‐inducing ligand (TRAIL)‐dependent mechanisms in IFN‐α/β‐dependent mechanisms.^149^, ^150^ Also, TLR7 is essential to stimulate IFN‐α/β‐produced pDCs,^151^ leading to TRAIL and granzyme B secretion via IFNAR1 signaling^152^ as depicted in Figure 4A. Tolerogenic pDCs in tumor infiltrates, on the other hand, are related to worse clinical outcomes because they resist tumor‐draining lymph nodes and surrounding solid tumors, as well as express high IDO.^3^, ^153^ In addition, coculturing ILC3s and pDCs increased the expression of apoptosis‐related genes caspase 3 and CD95.^139^ The addition of anti‐IFNα could degrade the effectiveness. Therefore, pDCs can trigger the apoptosis of ILC3s via the CD95 pathway, release IFN‐α in the tumor‐like microenvironment, and modulate the immune response against colon cancer, potentially improving the patient's prognosis.

#### 5.1.4 Limitations and perspective

It is critical to be aware of the limitations of using pDCs as a measure of tumor development, since pDCs are a heterogeneous population with diverse functional properties. Besides, a lack of standardized assessment protocols may introduce variability and limit the comparability of results across studies. Of course, we cannot ignore the deviation caused by the design of sample acquisition and inspection, together with the factors of concern and the lack of understanding of the mechanism. We should take these limitations into account when interpreting the prognostic value of pDC density in colon cancer patients. Further studies with larger sample sizes, standardized assessment methods, and a comprehensive analysis of confounding factors are warranted to validate and refine the prognostic significance of pDC density in colon cancer.

### 5.2 Intestinal infections

Considering that pathogen invasion usually occurs in intestinal infections, the interaction between the microbiota and the human immune system affects the appropriate pathogen response. pDCs have a protective role in limiting the bacterial load in the gut and helping to maintain the intestinal barrier (Figure 4B). Although pDCs have an essential immunomodulatory role in viral infections, little is known about their involvement in bacterial, parasitic, and viral infections.

#### 5.2.1 Gut microbiota affects pDCs’ functions

In a steady state, pDCs’ functions were regulated by cytokines. In a recent study, pDCs were verified as a main generator of the steady‐state type I IFN in response to the microbiota signals,^154^ due to the fact that pDCs mostly contribute to the distribution of IFN‐β in MLN and PP, which may refer to PRR signals. With regard to the influence of microbiota immunological responses, it is essential for homeostatic pDC distribution by triggering constitutive amounts of the chemokine CCL2, which is implicated in CCR2 signaling.^155^ CCR2 deficiency affects pDC transformation in the steady state, as pDC frequencies and quantities in the spleen and blood were decreased in Ccr2^−/−^ mice. It is impossible to ignore the importance of mononuclear phagocytes in the synthesis of CCL2 and CCR2 ligands due to their function in generating CCL2 primed by microbiota.^156^, ^157^ As demonstrated by injecting C57BL/6 mice with liposomes containing PBS or clodronate liposomes, which can deplete macrophage and eliminate mononuclear phagocytes, the CCR2^hi^ pDC subset accumulated in peripheral compartments, recruited by CCL2.^155^ To sum up, the gut microbiota has an impact on mononuclear phagocytes, which can trigger CCL2 expressions, affect pDC homeostatic transfer, and reveal novel targets for inflammation.

#### 5.2.2 Commensal microbes’ disorder

The gastrointestinal tract hosts a wide range of commensal species that coexist with the host and are known as commensal bacteria. Microbiota influence the microenvironment of the intestinal tract significantly, as they may preserve homeostasis while simultaneously shaping the immunity system. In this context, the gastrointestinal immune system must balance protective immune responses to potentially pathogenic microbes with immunologic nonresponsiveness to commensal flora and food ingredients, a process known as the gut‐resident DCs tolerance to commensal microbes.^17^, ^87^

It is unknown how gut microorganisms influence the pDC pool. *Bacteroides fragilis* represents a prototypical commensal bacteria. In earlier research, *B. fragilis* induces pDCs in MLNs during ongoing colitis,^158^ but they decrease in mice with a limited microbiota that differs from that of specific‐pathogen‐free animals.^159^ The ability of a microorganism to create pDCs in the small intestine and the colon was strongly correlated to the strains’ ability to increase colonic pDCs and overall FoxP3^+^ Treg frequencies, reflecting pDCs’ tolerogenic potential and ability to activate Tregs.^160^ There is a same effect in the *Bifidobacterium infantis* infection, which triggers the pDCs stimulation, induces T cell Foxp3^+^ expression, and secretes IL‐10 via the TLR‐9‐dependent pathway in response to the infection.^161^

In both the small intestine and the colon, the expression of a group of genes and IFN‐inducible signature transcripts correlates with pDC frequencies. *Il18* and *Tigit*'s transcripts are noteworthy. *Il18*, which is highly expressed by pDCs, inhibits the generation of type I IFN; and *Tigit*, a T cell marker, is substantially connected to the proportions of pDC and Treg.^160^ These experimental results give compelling evidence for us to pay close attention to intestinal flora and colonic pDC, particularly their mechanism and involvement in immune response, and to generate fresh therapeutic concepts for future therapeutic treatment.

#### 5.2.3 Pathogenic bacterial infections

Several studies have demonstrated the importance of the protective role of pDCs in bacterial invasion. pDCs have an essential immune regulatory function and modulate inflammation in the gut during *Citrobacter rodentium* infection. BDCA2‐DTR animals,^162^ along with pDCs deficiency, exposed to diphtheria toxin have a lower weight and increased bacteria load and inflammatory gene expression compared to *C. rodentium*‐infected mice. For instance, the level of mRNA expression of *Ifn‐γ, Il‐1*β, *Il22, Il‐17a*, and *tumor necrosis factor alpha (Tnf*α*)* ^127^, ^163^, ^164^, ^165^, ^166^ was significantly higher to protect against the infection.^166^ These evidences have corroborated that the bacteria and intestinal epithelium are more closely related, with some bacteria invading into intercellular space in the pDC‐depleted mice.

Overall, the richness of the gut microbiota is indeed bidirectional, emphasizing the need to maintain a healthy gut microbiota through intestinal microenvironment balance. A healthy gut environment, in turn, promotes the proliferation and diversity of beneficial bacteria, adding to the richness of the gut microbiota.

#### 5.2.4 Viral infections

As cytosolic DNA sensors, they recognize viral RNA; with receptor cooperation, they produce large quantities of type I IFN. Their critical role has been revealed in different types of virus infections.

Through the expansion of anti‐influenza virus T helper 1 (Th1) CD4^+^ T cells and cytotoxic T lymphocytes (CTLs), pDCs play an important role in the adaptive immune response against viruses.^167^ In vivo, pDCs target antigens through CD303 or CD367 to prime CD4^+^ T cells, proliferating and secreting a mixture of IL‐4 and IFN‐γ.^3^, ^144^, ^146^ It has also been corroborated that pDCs and type I IFN signaling contribute to B cell activation during the vivo intestinal rotavirus infection.^168^ Injection with αPDCA‐1 (CD137) antibody resulted in deletion of pDCs in the GALT, but had no effect on the B cell population. After 3 days of infection, there was a considerable rise in the PP and MLN of B cell activation treated with an isotype control antibody, which could be eliminated by αPDCA‐1 antibody.^168^ Besides, IFN‐α/β receptor signaling fully abrogated B cell activation in PPs and MLNs compared with SV129 wild‐type controls during murine rotavirus. Thus, during the infection, pDCs and type I IFN signaling are involved in B cell activation (Figure 4B). Moreover, the production of IFN‐β is in a TLR3‐ and TLR7‐dependent manner, protecting immunity during gut inflammation.^169^

It is common lore that pDCs play a significant role in human HIV/SIV infection; however, less is known about how they involved in the gastrointestinal mucosa immunity.^170^, ^171^ Notably, the amount of pDC in the peripheral blood decreased during chronic SIV infection,^172^ but the conversion of pDCs to the intestinal mucosa in the gastrointestinal mucosa was mainly at a high level, as indicated by higher levels of cell‐surface α4β7 on the cell surface, though the mechanism remains unknown. Furthermore, utilizing a three‐function intracellular cytokine labeling test, large frequencies of pDCs are positive for IFN‐α and macrophage inflammatory protein 1 (MIP‐1) expression but negative for TNF‐α, both of which are required to suppress HIV/SIV infection.^173^ According to the aforementioned phenomena, the frequency and cytokine secretion function of CD4^+^ pDCs in acute SIV infection are dramatically reduced as compared to the noninfected condition.^117^ It is noteworthy that enhancing CD4^+^ pDCs account for the bulk of those responsible for IFN‐α and TNF‐α production in response to TLR7/8 activation. Gut‐associated pDCs, in general, contribute to ongoing immunological activation, and their migration and accumulation in the intestinal mucosa are also important for the release of cytokines to combat infection. Furthermore, a group studied the blood of 29 HIV‐infected participants who started antiretroviral therapy during acute infection and underwent analytic treatment interruption. They discovered an increased frequency of pDCs before the detection of HIV RNA, as well as a decreased ability of pDCs to produce IFN‐α in vivo, which was correlated with decreased phosphorylation of IRF7 and NF‐κB.^174^ The reduced expression of *IFN* genes during analytic treatment stoppage was negatively associated with the delay in virus detection. As a result, blood testing for pDC frequency and function can be used to predict viral replication.

#### 5.2.5 Fungal infections

The collaboration of pDCs with cDCs, namely the recruitment of XCR1^+^ cDC1s that facilitate CD8^+^ T cell cross‐priming, may play a role in T cell cross‐priming and cross‐presentation. This is dependent on both TLR9 and type I IFN signaling, and inhibition of TLR9, MyD88, and NF‐κB at various phases in the innate response pathways (Figure 1) can prevent the activation of capsid‐specific CD8^+^ T cells during virus infection.^175^ Furthermore, the absence of IFN‐I‐producing pDC resulted in reduced steady‐state activation of CD8^+^ T cells by cDC. pDCs and microbiota provided tonic IFN‐I signals to cDCs in the steady state aiming to instruct the cDC basal metabolic state,^154^ and pDCs cooperated with cDCs to contribute to the priming of CD8^+^ T cells during infection, as shown in Figure 4B.^176^, ^177^

On top of that, in the intestinal microecological environment, compared with bacteria and viruses, the composition of fungi is unstable, and there are relatively few studies at present.^178^ Nowadays, research focused on *Saccharomyces cerevisiae*, a kind of *Saccharomyces* found in the gastrointestinal tract, has revealed its interaction with the host pDC.^179^ Once activated by SK‐1, pDCs uniquely sensed fungal nucleic acids, leading to the generation of a pDC subset P1‐pDCs (PD‐L1^+^ CD80^−^) to produce IFN‐α, and induce a Th profile producing IL‐10; however, it only weakly induced CD80 and CD86, as it was a typical response of CpG‐A retaining in the endosome and signaling through MyD88 and IRF‐7. Furthermore, degradation of ssRNA, ssDNA, and dsDNA of SK‐1 nucleic acids inhibits IFN‐α production by human pDCs. They did not, however, observe an additive impact on IFN‐a blocking owing to the TLR7 and TLR9 signaling inhibition. Interestingly, cDCs perform the opposite function of pDCs, including detecting cell wall components, expressing high levels of CD80, CD86, and IL‐6, and promoting a Th17 response. Interacting between pDCs and cDCs may facilitate a modest balance toward the host's homeostasis.

#### 5.2.6 Parasitic infections

pDCs’ effects vary depending on the different phases of infection. Here, we take parasite invasion as an example. pDCs are dispensable for Th2 response during the early stage of infection by *Schistosoma mansoni*.^46^ But levels of parasite‐specific IL‐4 and IL‐13 produced by MLN cells were analyzed to have a significant decrease as measured by ELISA, indicating a potential site‐specific role for pDCs in promoting the Th2 cell response. In later stages of infection, pDCs are essential for optimal Th2 cytokine production in response to *S. mansoni* eggs in the intestinal‐draining MLN and for egg‐specific IFN‐γ. Researchers discovered that pDCs and the infection recall response factors IL‐5, IL‐13, and IFN‐γ reduced in the MLNs at subsequent time points after using BDCA‐2‐DTR pDC‐depleted animals. In chronic infection, pDC depletion resulted in decreased Th2 cell cytokine production and activation in the liver. This sheds fresh light on the role of pDCs in various stages of the parasite infection response, which may be applied to a range of existing anti‐infective strategies.

In a word, the data show that pDCs play a unique role in the management of host microbiota‐immune system balance and anti‐fungal immune response, displaying a non‐negligible effect in targeting infections and inflammatory illnesses. Whereas the importance of pDCs in peripheral blood and the spleen has been well documented, less attention has been paid to pDCs in the gut mucosa. Without a doubt, investigating the underlying mechanisms of gut‐associated pDCs is vital to maintain the intestinal barrier against infection, which is critical to the onset, progression, and outcome of related disorders.

### 5.3 Role of gut pDCs in autoimmune diseases

A breakdown of intestinal homeostasis can result in IBDs, represented by Crohn's disease (CD) and ulcerative colitis (UC) in humans.^180^ The relationship between pDCs and autoimmune diseases in the gut is complex and context‐dependent.

#### 5.3.1 Epidemiology of IBD

The morbidity of IBD is witnessing an increase in both “low‐risk” and “high‐risk” countries, and nowadays it has become a global disease. Incidence of IBD patients in North America and Europe experienced the steepest increase toward the second half of the 20th century.^181^ The majority of patients are diagnosed early in life and the effect of IBD on health‐care systems will rise exponentially.^182^ However, little do we know about the pathogenesis of IBD.

#### 5.3.2 The inconsequential role of pDC in IBD

Genetic studies have indicated that pDCs play a potential role in a range of autoimmune diseases, including systemic lupus erythematosus and scleroderma (systemic sclerosis).^16^, ^17^ In contrast, pDCs do not play a major role in the pathogenesis of intestinal inflammation during IBD, they may have a minor role that arises upon destruction of the intestinal barrier by environmental factors.^183^ To address the role of pDCs in the initiation and progression of acute colitis, pDC‐ablated mice were given 2% DSS in drinking water for 7 days. They exhibited ameliorated clinical symptoms of acute colitis as compared with WT mice, whereas their reconstitution with WT pDCs restored this pathogenesis. They also targeted E2‐2 (TCF4) in experimental IBD caused by a deficiency of Wiskott‐Aldrich syndrome protein (WASP) or IL‐10. Tcf4 deletion, whether monoallelic or biallelic, had no effect on autoimmune symptoms in WASP‐deficient animals compared to SLE.^183^

In general, these findings imply that pDCs play a diverse role in autoimmune ailments, with immunogenic and tolerogenic functions depending on the context. While pDCs do not play a significant role in the etiology of IBD‐related intestinal inflammation, they may provide a limited function when environmental stimuli compromise the intestinal barrier.

#### 5.3.3 The pro‐inflammatory role of pDC in IBD

In various studies, pDCs are found to induce colitis by upregulating and downregulating the transcription of genes and pro‐inflammatory factors.

pDCs function as the primary and central cells, accumulating inflammatory phagocytes leading to the initiation and exacerbation of acute colitis. Research confirmed that pDCs accumulate in the inflamed colon upon mucosal injury. In a study investigating the pathophysiological roles of α7nAChRs on pDCs in the pathology of UC, pDC depletion significantly increased the survival rate of oxazolone (OXZ)‐induced colitis mice,^184^ as well as the severity and development of DSS‐induced colitis.^185^ In contrast, the deletion of pDCs is the amelioration of colitis and the mobilization of colitogenic phagocytes into the inflamed colon, which may be attributed to the disruption of abrogated mucosal CCL2 synthesis.^185^ However, further research is needed to fully understand the specific mechanisms underlying the relationship between disrupted CCL2 synthesis and the mobilization of colitogenic phagocytes.

Researchers revealed an association between pDC numbers in the inflamed colonic mucosa and MLN in IBD patients but a reduction in the periphery, which correlates with illness severity. Furthermore, CD40, CD86, TNF, IL6, and CXCL8 are reported to be overexpressed in IBD patients.^186^, ^187^ In CD mucosa, pDCs increased in the peripheral immune population and hence substantially expressed IL‐1β.^188^ It is also reported that the circulating pDCs migrate to secondary lymphatic organs and inflammatory sites in active IBD patients, secreting the inflammatory cytokines IL‐6, IL‐8, and TNF‐α, resulting in a pro‐inflammatory phenotype in Th1.^186^

IL‐36α was found to be overexpressed by intestinal epithelial cells, macrophages, CD8^+^ T cells, and/or pDCs in patients with active IBD.^189^ Further research is needed to explicitly understand the role of IL‐36α and its interaction with pDCs in autoimmune diseases in the gut.

We suppose that the reason for the different experimental results mentioned above is the inconsistent selection of experimental methods, specimens, and indicators. Therefore, the role of pDCs in IBD requires a comprehensive analysis of multiple surface markers, transcriptional factors, and genes to be determined.

Thus, the involvement of pDCs keeps vague, and it is worth exploring which character they play in IBD. Among the above results, pDCs themselves and the factors related to colitis may be attractive and effective cellular and molecular targets in IBD treatment therapy and improve prognosis for patients thereby.

### 5.4 Role of pDCs in intestinal graft‐versus‐host disease (GVHD)

GVHD is a severe immunological consequence of allogeneic hematopoietic stem cell transplantation (allo‐HSCT) that mediates graft‐versus‐leukemia (GVL) and graft‐versus‐tumor.^190^, ^191^ Typical GVHD, a potentially fatal condition, predominantly affects the skin, liver, and gastrointestinal tract.^190^ The gastrointestinal tract is the most common location of GVHD.

Clinical studies revealed that severe GVHD was associated with a decrease in the number of donor pDCs and unsuccessful post‐transplantation reconstitution.^192^, ^193^ This was accomplished by reducing DC rather than pDCs maturation. GVHD inhibited Flt3‐expressing donor MPPs, which was linked to Tcf4 suppression, a critical transcription factor in pDCs development.^194^ The ability of pDCs to stimulate virus‐specific T‐cell responses and antigen presentation via MHC II was impaired during GVHD in a clinical mouse model.^195^

Alloreactive T‐cell responses that mediate tissue injuries cause GVHD. As mentioned above, pDCs are responsible for the differentiation of the Th17 subset, which has been shown to play a role in acute GVHD in both mice and humans.^196^ In the disordered intestinal mucosa, the number of Th17 cells and CD123^+^ pDCs was significantly higher, which corresponded to the histological grade of acute GVHD. Moreover, CCR9^+^ pDCs, which are effective inducers of regulatory T‐cell activity, were recruited to the intestines via the CCR9 ligand CCL25, a gut‐homing chemokine of T cells, and pDCs. They suppressed acute GVHD induced by allogeneic CD4^+^ donor T cells by attenuating Ag‐specific immune responses in vitro and in vivo.^197^

Studies also demonstrated that donor pDCs regulated GVHD activities by restricting donor T‐cell activation via vasoactive intestinal polypeptide (VIP)‐receptor signaling by mobilization with Flt‐3L, which was beneficial in the prevention and treatment of GVHD through pDCs. Flt3L‐stimulated pDCs reduced GVHD while preserving GVL in both human and mouse leukemia following allo‐HSCT.^198^, ^199^ The gene expression patterns of Flt3L‐induced human and mouse pDCs are strikingly similar.^200^ Therefore, donor pDCs limited pathogenic T‐cell inflammation while also balancing GVHD and GVL activities.^191^

The essential role of pDCs has been validated in both humans and mice. Although clinical data hints that the transfer of donor‐type pDCs may be a cellular therapy for GVHD prevention,^194^ there is still a rigid demand for optimizing tolerogenic pDCs production therapy in GVHD, particularly how to manufacture adequate amounts of pDCs.

## 6 LIMINATION, FUTURE PERSPECTIVE, AND CHALLENGES

The primary goal of the review was to compile and combine novel information about the underlying activities of pDCs. To a certain extent, this large‐scale detailed evidence can be valuable in comprehending medical problems. However, we cannot ignore some of the limitations obtained from the adaptation data evidence.

First of all, based on a large number of previous data points, we believe that the limitations are that all the evidence comes from the knowledge of experimental animal models rather than human beings.^16^, ^18^ Second, in the cell experiment, the gene interference technique is utilized to further enhance the obtained phenomena; as a consequence, the technology ultimately gained is lowered, or a role of mutual compensation under particular overall conditions is neglected, resulting in a loss of integrity.^201^, ^202^, ^203^ Third, due to limited technology, it is difficult to collect total immune cells from infected intestinal tissue, making it unattainable to evaluate the Th2 and Treg responses in the MLN and LP.^46^

Of course, there are still certain issues to be addressed. On the one hand, it is unknown how pDC activation is regulated and how it influences aberrant situations that arise during infection in the current system of homeostasis.^204^ On the other hand, the underlying mechanisms of the immune response initiated in the intestine, as well as the interaction between pDCs and intestinal mucosa, require further investigation, and resolving these issues could lead to a novel strategy for the treatment of cancer infection and autoimmune‐related diseases.^119^

Within limits, we believe that the experimental evidence provides incredibly essential and valuable information for us to better understand the role of pDCs in clinical practice in the future. It is also worth mentioning that some of the findings in experimental animals are often congruent with those observed in humans. The same manipulation of the immune system, especially considering its own double‐edged sword effects on cultural importance, means that in some special cases, we must also use it to deal with more serious problems, especially in cancer since pDCs have complex concurrent impacts in different types of tumors.^143^ Of course, it is also worth noting that these research or knowledge gaps can be confirmed by other research methods, provide new areas for further research, constantly broaden our knowledge domains and methodologies, and provide new directions for the effective analysis of clinically related diseases in the future.

## 7 CONCLUSION

Based on the aforementioned summary, we may make the following deductions: to begin with, pDCs are specialized DCs, and single‐cell transcription is utilized to analyze cells. The complicated demands of pDCs’ development and function for many tumor factors are progressively becoming obvious, and their significance in numerous domains, including cancer, infections, and autoimmune illnesses, is increasingly becoming clear. Second, we emphasize pDC's function in intestinal mucosal immunity. Despite certain gaps in current research, evidence from numerous studies indicates that gut‐associated pDCs have essential roles in the intestinal mucosa, namely in the formation of type I interferon‐responsive Treg and Th cells that have engaged in immunological responses. Furthermore, we highlight that pDCs may protect the host against a wide range of pathogens from invasion to defense, providing strong evidence for rational therapeutic intervention. More study in the treatment of immunodeficiency is clearly needed to supplement the current therapeutic approaches.

## AUTHOR CONTRIBUTIONS

Xueran Guo and Boya Wang wrote the draft. Sitian Zhang and Chengwei He polished the figures. Han Gao, Shuzi Xin, and Xiaohui Liu analyzed the data from the literatures. Fengrong Gong, Xinyi Yu, and Luming Pan drew the graph and expanded the literature. Fanglin Sun and Jingdong Xu analyzed all the data and revised the manuscript. All authors have discussed the review and approved for the final version and agree to be accountable for all aspects of the work.

## CONFLICT OF INTEREST STATEMENT

The authors declare no conflict of interest.
